# Impact of pathophysiologically relevant underlying disease burden on pathogen distribution and antimicrobial resistance in patients with device-associated urinary tract infections

**DOI:** 10.3389/fcimb.2026.1822210

**Published:** 2026-07-03

**Authors:** Yue Jiang, Zehua Du, Liping Wang

**Affiliations:** Department of Infectious Diseases, The Affiliated Hospital of Xuzhou Medical University, Xuzhou, Jiangsu, China

**Keywords:** antimicrobial resistance, *Escherichia coli*, indwelling medical device, underlying disease burden, urinary tract infection

## Abstract

**Objective:**

To investigate the impact of pathophysiologically relevant underlying disease burden on the distribution of pathogenic bacteria and antimicrobial resistance in patients with device-associated urinary tract infections (DA-UTIs), and to provide evidence for precise empirical therapy and infection prevention and control in high-risk populations.

**Methods:**

Only underlying diseases with known pathophysiological relevance to urinary tract infection (e.g., diabetes mellitus, urinary stones/obstruction, renal insufficiency, etc.) were considered. A retrospective study was conducted on 449 patients with DA-UTIs admitted to the Affiliated Hospital of Xuzhou Medical University from January 2020 to December 2024. Patients were stratified into three groups according to the number of underlying diseases: Group A (no underlying diseases), Group B (one underlying disease), and Group C (≥2 underlying diseases). Clinical manifestations and pathogenic spectrum composition were compared among the three groups, and the antimicrobial resistance characteristics of *Escherichia coli* and *Klebsiella pneumoniae* were analyzed.

**Results:**

The median age of the patients was 66 years, and most patients were admitted to the Department of Urology and Intensive Care Unit (ICU). *Escherichia coli* was the predominant pathogen (41.6%), and the isolation rate of *Candida* species showed an increasing trend with the aggravation of underlying disease burden. Antimicrobial resistance analysis revealed a significant correlation between underlying disease burden and the resistance of *Escherichia coli* to fluoroquinolones: Group C had a significantly increased risk of ciprofloxacin resistance (*P* = 0.009), while Group B had a higher resistance risk to levofloxacin (*P* = 0.018) and norfloxacin (*P* = 0.019). For *Klebsiella pneumoniae*, the resistance risks to cefotetan (*P* = 0.029), doripenem (*P* = 0.036), and amikacin (*P* = 0.026) were significantly elevated in Group B. Carbapenems, aminoglycosides, and polymyxins maintained high susceptibility across all groups.

**Conclusion:**

In patients with DA-UTIs, pathophysiologically relevant underlying disease burden is closely associated with antimicrobial resistance of pathogenic bacteria, particularly significantly increasing the risk of fluoroquinolone resistance. Routine use of fluoroquinolones should be avoided in such high-risk patients for clinical empirical therapy. The initial treatment regimen should be optimized and etiological testing should be strengthened based on local antimicrobial resistance surveillance data and the patient’s comorbidity status.

## 1 Introduction

Urinary tract infection (UTI), one of the most common bacterial infectious diseases in clinical practice, continues to impose a substantial burden on public health systems and individual health worldwide (Flores-Mireles et al., 2015; Yang et al., 2022; Global burden of bacterial antimicrobial resistance 1990-2021: a systematic analysis with forecasts to 2050, 2024). Indwelling medical devices (e.g., urinary catheters, ureteral stents), as essential tools in the diagnosis and treatment of urinary system diseases and critical care support (Yao et al., 2022; Sleziak et al., 2025), significantly increase the risk of complicated urinary tract infections (cUTIs) while playing a pivotal clinical role (He et al., 2024). Existing studies have fully confirmed that the pathogenic spectrum of device-associated urinary tract infections (DA-UTIs) possesses unique characteristics, and the detection rate of multidrug-resistant (MDR) bacteria is significantly higher than that of community-acquired infections (Ehdaie et al., 2021), thereby rendering clinical treatment more challenging. Among the numerous clinical challenges, the most difficult task in patients with indwelling urinary devices is differentiating symptomatic urinary tract infection from asymptomatic bacteriuria. Current clinical guidelines do not recommend antibiotic treatment for asymptomatic bacteriuria, as this practice increases the risk of Clostridioides difficile colitis and accelerates the dissemination of antimicrobial-resistant bacteria (Barlam et al., 2016). Furthermore, device-associated urinary tract infections (DA-UTIs) are frequently polymicrobial in nature, and biofilms in patients with long-term indwelling catheters can harbor multiple bacterial and fungal pathogens. This polymicrobial environment makes the choice of empirical antibiotic therapy highly unpredictable (Kohler et al., 2026).

In this study, we considered only underlying diseases with established pathophysiological relevance to urinary tract infection (e.g., diabetes mellitus, urinary stones/obstruction, renal insufficiency, etc.), as detailed in the Methods section.

A prominent dilemma in current clinical practice is the heterogeneity of infectious pathogens and drug resistance patterns among patients facing similar risks of indwelling device placement. Previous studies have mostly focused on the impact of a single type of underlying disease (e.g., diabetes mellitus (Ramrakhia et al., 2020), chronic kidney disease (Dimitrijevic et al., 2021)) or a single indwelling device (Scotland et al., 2019)on the development of UTIs. However, in real-world clinical practice, patients often suffer from multiple chronic diseases simultaneously. This comorbid state, which leads to cumulative impairment of physiological functions, alterations in immune status, and repeated medical interventions (Alhazmi et al., 2023), may jointly form a unique host microenvironment that is susceptible to infection and prone to the selection of drug-resistant bacteria. Therefore, regarding the patient’s underlying disease burden (i.e., the number of comorbid diseases) as a comprehensive and generalized risk assessment index beyond a single disease diagnosis to predict the complexity of infection, the tendency of pathogenic bacteria to develop drug resistance, and treatment response holds important theoretical and practical value. To fill this critical research gap, the present study adopted a stratified framework centered on the number of underlying diseases, aiming to systematically explore whether the composition of pathogenic bacteria and antimicrobial resistance characteristics of UTIs in patients with indwelling medical devices undergo significant and regular changes with the increase in the number of comorbid underlying diseases. This study is expected to provide key evidence for more precise treatment of UTIs, optimize the allocation of medical resources, and improve the clinical outcomes of patients with complex comorbidities.

## 2 Materials and methods

### 2.1 Study participants and data sources

Data were extracted from the electronic medical record system of the Affiliated Hospital of Xuzhou Medical University. This study adopted a retrospective design. Patients hospitalized between January 1, 2020, and December 31, 2024, who were diagnosed with urinary tract infection were screened in accordance with predefined inclusion and exclusion criteria. A total of 449 eligible patients were ultimately enrolled in the study.

#### 2.1.1 Inclusion criteria

(1) Age ≥ 18 years; (2) Positive midstream urine culture results with etiological diagnosis consistent with the established criteria (Nelson et al., 2024); (3) Complete medical records and laboratory examination data available; (4) Presence of iatrogenic foreign bodies in the urinary tract, including indwelling urinary catheters, ureteral stents, or receipt of intermittent catheterization.

#### 2.1.2 Exclusion criteria

(1) Incomplete clinical data; (2) Negative urine culture results, or isolation of ≥ 3 bacterial species from clean midstream urine culture (Nelson et al., 2024).

### 2.2 Collection of general clinical data

(1) Demographic data: age, gender, admitting department; (2) Clinical features: symptoms (fever, lumbago, urinary irritation symptoms), physical signs (costovertebral angle tenderness); (3) Underlying diseases: The underlying diseases included in the analysis were common types with definite pathophysiological significance for UTIs, including increased residual urine; obstructive uropathy caused by any reason (e.g., bladder outlet obstruction, neurogenic bladder, urinary calculi, tumors, benign prostatic hyperplasia) (Tolani et al., 2020); vesicoureteral reflux or other urinary tract dysfunction; urinary diversion or other anatomical abnormalities (e.g., urethrovaginal fistula, ureteroenteric fistula); uroepithelial injury caused by chemotherapy or radiotherapy; perioperative and postoperative periods; renal insufficiency; diabetes mellitus; and prolonged bed rest due to cerebrovascular diseases etc (Xie et al., 2023). Patients with any of the above diseases were identified as having underlying diseases.

### 2.3 Laboratory data

Antimicrobial susceptibility testing was performed using the broth microdilution method to determine minimum inhibitory concentrations (MICs), and results were interpreted according to CLSI guidelines (most current edition used in our laboratory). Quality control strains included *Escherichia coli* ATCC 25922, *Pseudomonas aeruginosa* ATCC 27853, and Staphylococcus aureus ATCC 29213.

### 2.4 Imaging data

Urinary system color Doppler ultrasound, abdominal computed tomography (CT), and abdominal magnetic resonance imaging (MRI) were reviewed. These imaging data were used to exclude other urinary system lesions that might affect the diagnosis of UTIs.

### 2.5 Definition of device-associated urinary tract infection

According to the EAU Guidelines on Urological Infections, a device-associated urinary tract infection (DA-UTI) refers to a urinary tract infection occurring in a patient with an indwelling urinary device (including urethral catheter, ureteral stent, nephrostomy tube, or suprapubic catheter) or within 48 hours after device removal. Symptomatic infection is defined as a positive urine culture accompanied by urinary or systemic symptoms that cannot be explained by other causes, distinguishing it from asymptomatic bacteriuria.

### 2.6 Grouping criteria

To systematically evaluate the impact of underlying diseases on the clinical characteristics of UTIs, patients were stratified according to the presence of indwelling urinary devices and the status of underlying diseases: Group A (no underlying diseases as mentioned above); Group B (one underlying disease); Group C (≥2 underlying diseases).

### 2.7 Ethical approval

This study was approved by the Ethics Committee of the Affiliated Hospital of Xuzhou Medical University (Approval No.: XYFY2025-KL449-01). All procedures were conducted in accordance with the Declaration of Helsinki.

### 2.8 Statistical analysis

Statistical analysis was performed using SPSS software (Version 27.0). Measurement data were tested for normality using the Kolmogorov-Smirnov test and Shapiro-Wilk test. Normally or approximately normally distributed data were expressed as mean ± standard deviation, while skewed distributed data were presented as median (interquartile range). The Mann-Whitney U test was used for comparison between two groups, and the Kruskal-Wallis H test was applied for multiple group comparisons. Count data were expressed as number (n) and percentage (%). The chi-square test was used for comparison of rates between groups after evaluating the applicability conditions; the Fisher’s exact probability test was adopted when the theoretical frequency of more than 20% of cells in the contingency table was less than 5. Binary logistic regression analysis was used to calculate the odds ratio (OR) and 95% confidence interval (95% CI) for analyzing the impact of underlying disease burden on antimicrobial resistance. A two-sided *P*-value < 0.05 was considered statistically significant.

### 3 Results

### 3.1 Gender and age distribution of patients

In this study, symptomatic DA-UTI was diagnosed according to the EAU guidelines as defined in the Methods, and all enrolled patients had asymptomatic bacteriuria excluded. A total of 449 patients with DA-UTIs were enrolled during the 5-year study period (2020–2024). Among them, 218 (48.6%) were male and 231 (51.4%) were female. The median age of all patients was 66.0 years, and the age of male patients was significantly higher than that of female patients (68.0 vs. 64.0 years, *P* = 0.004) (**Table 1**). According to the number of underlying diseases, 130 patients (29.0%) were assigned to Group A, 226 (50.3%) to Group B, and 93 (20.7%) to Group C. There were no significant differences in gender composition and age distribution among the three groups (*P*>0.05) (**Table 2**).

**Table 1:** Gender, age distribution and number of isolated bacterial strains in all patients [n (%)].

Group	Case number	Age (years)	Z value	P value	Number of isolated strains
Total	449	66.0 (54.0, 76.0)	-	-	488
Male	218 (48.6)	68.0 (56.0, 77.0)	-2.856	0.004	237 (48.6)
Female	231 (51.4)	64.0 (52.0, 75.0)	-	-	251 (51.4)

**Table 2:** Gender and age distribution of patients in different groups [n (%)].

Group	Case number	Male	Female	χ²	P-value	Age (years)	H value	P-value
Group A	130 (29.0)	66 (50.8)	64 (49.2)	1.186	0.553	67.5 (53.0, 77.0)	5.731	0.057
Group B	226 (50.3)	104 (46.0)	122 (54.0)	-	-	64.0 (52.8, 75.0)	-	-
Group C	93 (20.7)	48 (51.6)	45 (48.4)	-	-	69.0 (60.0, 76.0)	-	-

χ², Chi-square statistic.

### 3.2 Composition of underlying diseases in different groups

In Group B, the most common single comorbidity was urinary stones/obstructive disease (37.2%), followed by diabetes mellitus (33.2%) and malignancy (16.8%). In Group C (≥2 underlying diseases), diabetes mellitus (75.3%) and urinary stones/obstructive disease (72.0%) were the two most common comorbidities, which frequently coexisted (**Table 3**).

**Table 3:** Composition of underlying diseases in different groups [n (%)].

Underlying disease	Group B (n=226)	Group C (n=93)
Diabetes mellitus	75 (33.2)	70 (75.3)
Urinary stones/obstructive disease	84 (37.2)	67 (72.0)
Malignancy	38 (16.8)	18 (19.4)
Prolonged bed rest	3 (1.3)	4 (4.3)
Structural/functional urinary abnormality	11 (4.9)	19 (20.4)
Renal insufficiency	5 (2.2)	20 (21.5)
Kidney transplantation	2 (0.9)	1 (1.1)
Autoimmune disease	8 (3.5)	1 (1.1)

Patients in Group C could have more than one underlying disease; therefore percentages sum to >100%.

### 3.3 Department distribution

**Figure 1:**
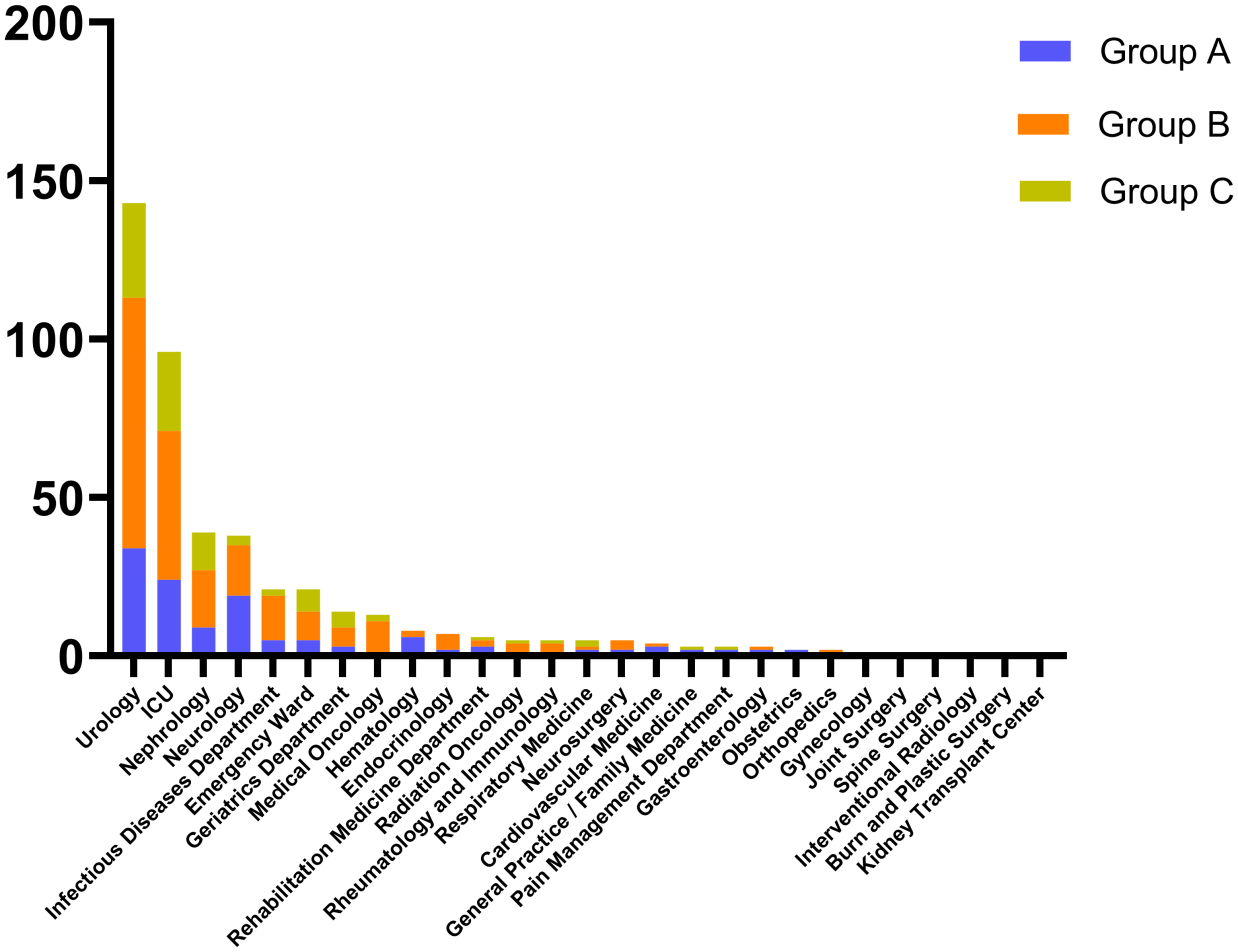
Department distribution of the enrolled patients.

Among all patients, the top four admitting departments were the Department of Urology (31.8%), ICU (21.4%), Department of Nephrology (8.7%), and Department of Neurology (8.5%). Group A was mainly distributed in the Department of Urology (26.2%), ICU (18.5%), Department of Neurology (14.6%), and Department of Nephrology (6.9%). Group B was more concentrated in the Department of Urology (35.0%), ICU (20.8%), Department of Nephrology (8.0%), and Department of Neurology (7.1%). The main admitting departments of Group C were the Department of Urology (32.3%), ICU (26.9%), Department of Nephrology (12.9%), and Emergency Ward (7.5%) (**Figure 1**).

### 3.4 Analysis of clinical symptoms

It is well recognized that patients with indwelling urinary catheters or stents often lack typical urinary irritative symptoms such as frequency, urgency, and dysuria. Therefore, in the present study, the diagnosis of symptomatic infection was not solely dependent on these local symptoms. Instead, it was based on a broader clinical assessment that also incorporated systemic signs, particularly fever, in accordance with the EAU guideline definition described in the Methods (Section 3.3). There were no statistically significant differences in the incidence of fever, urinary irritation symptoms (including frequent micturition, urgent micturition, dysuria) and lumbago (including lumbosacral soreness, low back pain) among the three groups (*P*> 0.05). The incidence of fever (35.4% in Group A, 38.1% in Group B, 41.9% in Group C) and lumbago (5.4% in Group A, 11.9% in Group B, 10.8% in Group C) showed a numerical increasing trend with the aggravation of underlying disease burden, but the differences were not statistically significant (**Table 4**).

**Table 4:** Analysis of clinical symptoms in different groups [n (%)].

Group	Fever	Urinary irritation symptoms	Lumbago
Group A	46 (35.4)	24 (18.5)	7 (5.4)
Group B	86 (38.1)	40 (17.7)	27 (11.9)
Group C	39 (41.9)	19 (20.4)	10 (10.8)
χ²	0.987	0.326	4.141
P-value	0.611	0.850	0.126

χ², Chi-square statistic.

### 3.5 Comparison of pathogenic spectrum of urinary tract infections

In patients with long-term indwelling catheters or stents, typical urinary irritative symptoms are often absent. In this study, according to the EAU guideline definition (see Methods), symptomatic infection was defined by the presence of either urinary or systemic symptoms, not limited to local irritative manifestations; therefore, patients without typical symptoms but with systemic signs (e.g., fever) were still included in the pathogen spectrum analysis.

**Figure 2:**
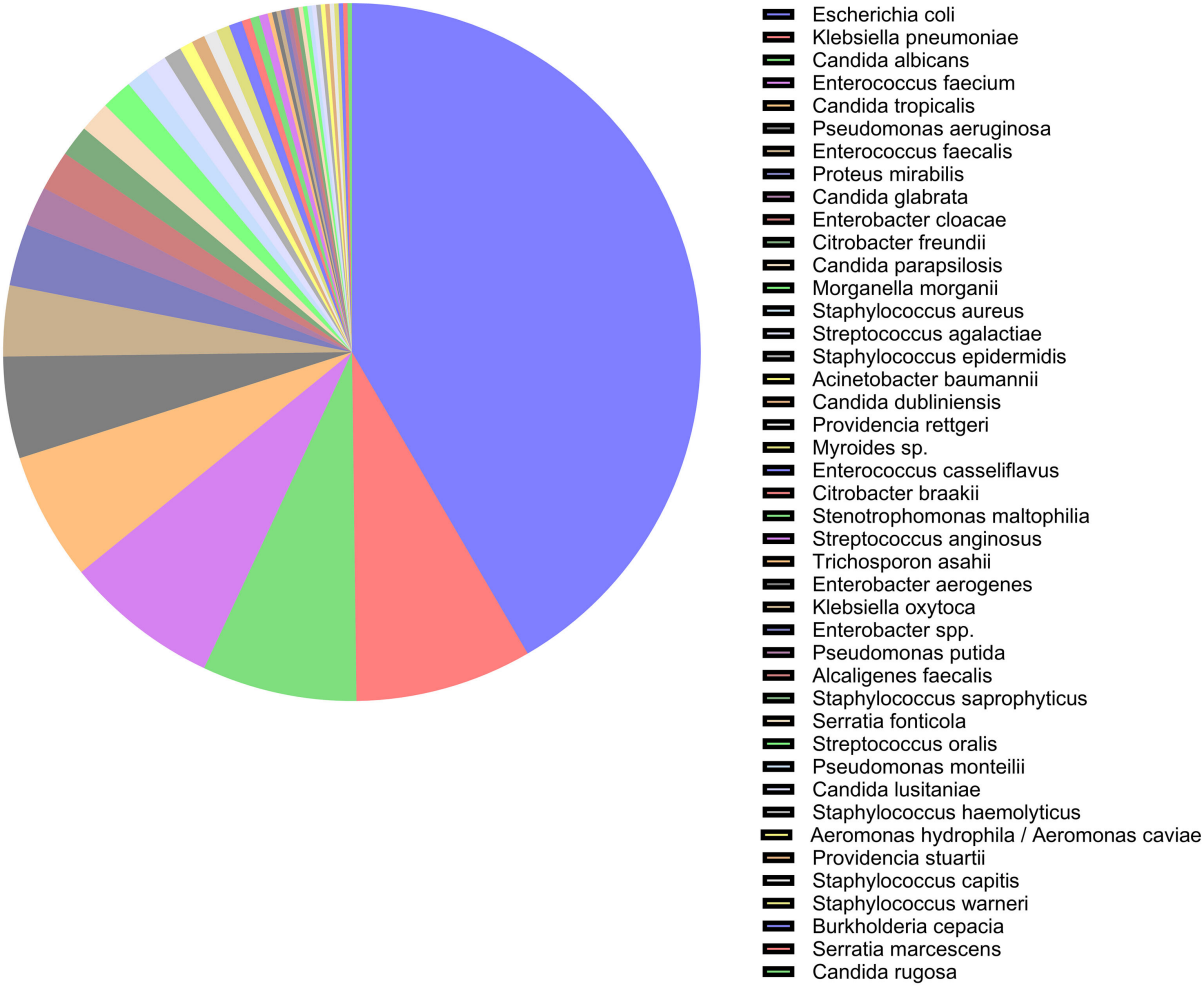
Distribution of pathogenic spectrum of urinary tract infections in all patients.

A total of 488 strains of pathogenic bacteria were isolated from the enrolled patients. *Escherichia coli* was the most predominant pathogen, accounting for 41.6% of all isolated strains, and its constituent ratio showed no significant difference among the three groups (39.3% in Group A, 42.6% in Group B, 42.3% in Group C, *P* = 0.804). Other common pathogens included *Klebsiella pneumoniae* (8.2%), *Candida albicans* (7.2%) and *Enterococcus faecium* (7.2%). Notably, the isolation rate of *Candida* species (including *Candida albicans* and other *Candida* species) presented an increasing trend with the aggravation of underlying disease burden (13.6% in Group A, 18.0% in Group B, 22.1% in Group C). Chi-square test showed no statistically significant differences in the constituent ratios of the above pathogens among the three groups (*P*> 0.05) (**Figure 2**, **Table 5**).

**Table 5:** Analysis of pathogenic bacteria distribution in different groups [n (%)].

Group	*Escherichia coli*	*Klebsiella pneumoniae*	*Enterococcus faecium*	Candida albicans	Candida species
Group A	55 (39.3)	12 (8.6)	13 (9.3)	9 (6.4)	19 (13.6)
Group B	104 (42.6)	22 (9.0)	16 (6.6)	16 (6.6)	44 (18.0)
Group C	44 (42.3)	6 (5.8)	6 (5.8)	10 (9.6)	23 (22.1)
χ²	0.435	1.058	1.385	1.187	3.057
P-value	0.804	0.589	0.500	0.552	0.217

### 3.6 Correlation analysis between underlying disease burden and antimicrobial resistance

#### 3.6.1 Antimicrobial resistance analysis of *Escherichia coli*

Underlying disease burden was an important risk factor for predicting the resistance of *Escherichia coli* to fluoroquinolones. Compared with Group A, Group C (≥2 underlying diseases) had a significantly increased risk of resistance to ciprofloxacin, levofloxacin, moxifloxacin and norfloxacin (*P* < 0.05), among which the risk of ciprofloxacin resistance was 7.7-fold higher (OR = 7.69, 95%CI: 1.65–35.80, *P* = 0.009). In addition, for levofloxacin and norfloxacin, even patients with only one underlying disease (Group B) had a significantly elevated resistance risk (*P* < 0.05). Among cephalosporins, only the resistance risk to ceftizoxime in Group C was significantly higher than that in Group A (OR = 3.20, 95%CI: 1.20–8.55, *P* = 0.021). There were no significant differences in the resistance rates of carbapenems, aminoglycosides, polymyxins and tigecycline among the three groups (**Table 6**).

**Table 6:** Antimicrobial resistance of *Escherichia coli* and *Klebsiella pneumoniae* in different groups [n (%)].

*Escherichia coli*	Group A	Group B	Group C	χ² (P-value)	Binary logistic regression (OR[95%CI], P-value)	Binary logistic regression (OR[95%CI], P-value)	*Klebsiella pneumoniae*	Group A	Group B	Group C	P-value	Binary logistic regression (OR[95%CI], P-value)	Binary logistic regression (OR[95%CI], P-value)
Cefalotin (n=163)	38 (79.2)	67 (84.8)	30 (83.3)	0.677 (0.713)	-	-	Cefalotin (n=33)	9 (90.0)	16 (84.2)	2 (50.0)	0.261*	-	-
Cefuroxime (n=165)	37 (77.1)	65 (81.3)	31 (83.8)	0.641 (0.726)	-	-	Cefuroxime (n=33)	8 (80.0)	16 (84.2)	2 (50.0)	0.324*	-	-
Cefuroxime axetil (n=163)	37 (77.1)	62 (78.5)	30 (83.3)	0.527 (0.768)	-	-	Cefuroxime axetil (n=33)	8 (80.0)	16 (84.2)	2 (50.0)	0.324*	-	-
Cefotetan (n=163)	7 (14.6)	4 (5.1)	2 (5.6)	0.177*	-	-	Cefotetan (n=33)	1 (10.0)	11 (57.9)	1 (25.0)	0.028*	B vs A: 12.375 (1.294, 118.331), 0.029	C vs A: 3.000 (0.140, 64.262), 0.482
Cefotaxime (n=163)	36 (75.0)	63 (79.7)	30 (83.3)	0.899 (0.638)	-	-	Cefotaxime (n=33)	7 (70.0)	15 (78.9)	2 (50.0)	0.478*	-	-
Ceftazidime (n=201)	27 (49.1)	57 (55.3)	26 (60.5)	1.292 (0.524)	-	-	Ceftazidime (n=40)	7 (58.3)	16 (72.7)	4 (66.7)	0.728*	-	-
Cefoperazone/sulbactam (n=200)	7 (12.7)	10 (9.8)	4 (9.3)	0.409 (0.815)	-	-	Cefoperazone/sulbactam (n=40)	4 (33.3)	12 (54.5)	3 (50.0)	0.461*	-	-
Cefpodoxime (n=163)	37 (77.1)	65 (82.3)	30 (83.3)	0.689 (0.709)	-	-	Cefpodoxime (n=32)	8 (80.0)	15 (83.3)	2 (50.0)	0.395*	-	-
Ceftizoxime (n=155)	12 (52.3)	48 (64.0)	28 (77.8)	5.583 (0.061)	B vs A: 1.623 (0.762, 3.459), 0.209	C vs A: 3.196 (1.195, 8.545), 0.021	Ceftizoxime (n=32)	6 (66.7)	14 (73.7)	2 (50.0)	0.746*	-	-
Cefepime (n=199)	26 (47.3)	59 (58.4)	28 (65.1)	3.354 (0.187)	-	-	Cefepime (n=40)	9 (75.0)	15 (68.2)	4 (66.7)	1.000*	-	-
Doxycycline (n=199)	27 (49.1)	52 (51.0)	21 (50.0)	0.052 (0.974)	-	-	Doxycycline (n=40)	9 (75.0)	16 (72.7)	3 (50.0)	0.570*	-	-
Amoxicillin/clavulanic acid (n=163)	14 (29.2)	21 (26.6)	17 (47.2)	5.084 (0.079)	-	-	Amoxicillin/clavulanic acid (n=33)	5 (50.0)	14 (73.7)	1 (25.0)	0.135*	-	-
Imipenem (n=201)	3 (5.5)	3 (2.9)	1 (2.3)	0.600*	-	-	Imipenem (n=39)	3 (25.0)	10 (47.6)	2 (33.3)	0.493*	-	-
Meropenem (n=201)	3 (5.5)	3 (2.9)	1 (2.3)	0.600*	-	-	Meropenem (n=40)	3 (25.0)	10 (45.5)	2 (33.3)	0.496*	-	-
Nalidixic acid (n=135)	36 (100.0)	63 (96.9)	33 (97.1)	0.615*	-	-	Nalidixic acid (n=25)	7 (77.8)	13 (86.7)	0 (0.0)	0.217*	-	-
Piperacillin (n=162)	45 (93.8)	72 (92.3)	35 (97.2)	0.639*	-	-	Piperacillin (n=32)	1 (100.0)	17 (89.5)	3 (75.0)	0.494*	-	-
Tetracycline (n=163)	33 (68.8)	57 (72.2)	22 (61.1)	1.402 (0.496)	-	-	Tetracycline (n=33)	9 (90.0)	14 (73.7)	2 (50.0)	0.256*	-	-
Ticarcillin/clavulanic acid (n=199)	14 (25.5)	28 (27.5)	16 (38.1)	2.134 (0.344)	-	-	Ticarcillin/clavulanic acid (n=39)	7 (58.3)	14 (66.7)	4 (66.7)	0.900*	-	-
Trimethoprim-sulfamethoxazole (n=201)	36 (65.5)	65 (63.1)	31 (72.1)	1.088 (0.580)	-	-	Trimethoprim-sulfamethoxazole (n=39)	7 (58.3)	16 (76.2)	2 (33.3)	0.163*	-	-
Doripenem (n=136)	3 (8.6)	2 (3.0)	1 (2.9)	0.547*	-	-	Doripenem (n=25)	1 (11.1)	9 (60.0)	0 (0.0)	0.034*	B vs A: 12.000 (1.178, 122.274), 0.036	-
Minocycline (n=198)	14 (25.9)	33 (32.4)	16 (38.1)	1.640 (0.440)	-	-	Minocycline (n=40)	8 (66.7)	14 (63.6)	2 (33.3)	0.412*	-	-
Amikacin (n=200)	7 (12.7)	17 (16.7)	7 (16.3)	0.449 (0.799)	-	-	Amikacin (n=40)	2 (16.7)	13 (59.1)	2 (33.3)	0.041*	B vs A: 7.222 (1.268, 41.143), 0.026	C vs A: 2.500 (0.256, 24.375), 0.430
Ciprofloxacin (n=201)	40 (72.7)	88 (85.4)	41 (95.3)	9.517 (0.009)	B vs A: 2.200 (0.981, 4.933), 0.056	C vs A: 7.687 (1.651, 35.800), 0.009	Ciprofloxacin (n=40)	9 (75.0)	18 (81.8)	5 (83.3)	0.864*	-	-
Levofloxacin (n=200)	38 (69.1)	87 (85.3)	40 (93.0)	10.699 (0.005)	B vs A: 2.595 (1.175, 5.729), 0.018	C vs A: 5.965 (1.617, 22.001), 0.007	Levofloxacin (n=40)	8 (66.7)	18 (81.8)	5 (83.3)	0.581*	-	-
Moxifloxacin (n=163)	33 (68.8)	66 (83.5)	33 (91.7)	7.668 (0.022)	B vs A: 2.308 (0.984, 5.411), 0.054	C vs A: 5.000 (1.322, 18.909), 0.018	Moxifloxacin (n=33)	7 (70.0)	15 (78.9)	2 (50.0)	0.478*	-	-
Norfloxacin (n=162)	31 (64.6)	65 (83.3)	31 (86.1)	7.795 (0.020)	B vs A: 2.742 (1.185, 6.347), 0.019	C vs A: 3.400 (1.115, 10.363), 0.031	Norfloxacin (n=33)	7 (70.0)	15 (78.9)	2 (50.0)	0.478*	-	-
Piperacillin/tazobactam (n=200)	14 (25.5)	21 (20.6)	12 (27.9)	1.062 (0.588)	-	-	Piperacillin/tazobactam (n=39)	7 (58.3)	15 (68.2)	2 (40.0)	0.418*	-	-
Ticarcillin (n=164)	45 (93.8)	76 (95.0)	35 (97.2)	0.540 (0.764)	-	-	Ticarcillin (n=25)	8 (100.0)	15 (100.0)	2 (100.0)	-	-	-
Tobramycin (n=199)	22 (40.0)	40 (39.2)	25 (58.5)	5.414 (0.067)	-	-	Tobramycin (n=40)	4 (33.3)	15 (68.2)	3 (50.0)	0.168*	-	-
Aztreonam (n=199)	29 (52.7)	66 (64.7)	29 (69.0)	3.212 (0.201)	-	-	Aztreonam (n=40)	8 (66.7)	17 (77.3)	4 (66.7)	0.700*	-	-
Polymyxin (n=197)	0 (0.0)	1 (1.0)	1 (1.0)	0.459*	-	-	Polymyxin (n=38)	0 (0.0)	2 (9.1)	1 (20.0)	0.229*	-	-
Tigecycline (n=197)	0 (0.0)	0 (0.0)	0 (0.0)	-	-	-	Tigecycline (n=27)	1 (11.1)	2 (15.4)	0 (0.0)	1.000*	-	-

*Fisher’s exact probability test.

#### 3.6.2 Antimicrobial resistance analysis of *Klebsiella pneumoniae*

Underlying disease burden was also associated with the antimicrobial resistance of *Klebsiella pneumoniae* to specific antibiotics. Compared with patients without underlying diseases (Group A), the isolated strains from patients with one underlying disease (Group B) had a significantly increased risk of resistance to cefotetan (OR = 12.38, 95%CI: 1.29–118.33), doripenem (OR = 12.00, 95%CI: 1.18–122.27) and amikacin (OR = 7.22, 95%CI: 1.27–41.14) (*P* < 0.05). Notably, carbapenems (imipenem, meropenem) showed a relatively high resistance rate (25.0%–47.6%) in all groups, but there was no statistically significant difference among the groups (*P*>0.05). In addition, there were no significant differences in the resistance rates of other tested antibiotics among the groups with different underlying disease burdens, including fluoroquinolones (ciprofloxacin, levofloxacin, etc.), most cephalosporins (cefotaxime, ceftazidime, cefepime, etc.), aztreonam, piperacillin/tazobactam and tigecycline (*P*>0.05) (**Table 6**).

## 4 Discussion

Urinary tract infection (UTI) is one of the most common bacterial infections worldwide. In recent years, the pathogenic spectrum of UTI has evolved. Simultaneously, antimicrobial resistance has risen rapidly, making UTI a severe public health problem. Microbial identification technology has been widely applied, greatly improving the etiological diagnosis of UTIs. Antimicrobial resistance surveillance networks have also continuously improved, providing critical support for clinical therapy. However, complicated urinary tract infections (cUTIs) remain a highly heterogeneous disease (Mo et al., 2025). Their clinical manifestations, pathogenic composition, and drug resistance characteristics are often significantly affected by host factors, such as comorbid diseases and indwelling medical devices (Ling et al., 2023; Hari et al., 2024; Kranz et al., 2024; Nelson et al., 2024). Numerous clinical guidelines provide references for empirical anti-infective therapy. Nevertheless, core challenges persist in clinical practice. Complex underlying diseases can drive changes in antimicrobial resistance patterns. Furthermore, individual responses to treatment are often unpredictable. For patients with device-associated urinary tract infections (DA-UTIs), systematically evaluating the impact of underlying disease burden on infection characteristics is therefore crucial. Such evaluation holds great significance for promoting precise treatment and effective infection control in this population. To explore this issue in depth, the present study adopted a retrospective analysis method to systematically sort out the clinical data of 449 patients with a definite diagnosis of DA-UTIs with indwelling medical devices. Patients were stratified according to the number of comorbid underlying diseases: Group A (no underlying diseases), Group B (one underlying disease), and Group C (≥2 underlying diseases). This stratification aimed to systematically reveal the profound impact of underlying disease burden on patients’ clinical manifestations, pathogenic distribution characteristics, and antimicrobial resistance.

The baseline characteristics of the study population clearly outline the typical features of DA-UTIs. The median age of all patients was 66 years, and the age of male patients was significantly higher than that of female patients. This demographic characteristic is highly consistent with the epidemiological distribution of urinary system diseases and critical illnesses (Linhares et al., 2013). Elderly male patients are prone to lower urinary tract obstruction or micturition dysfunction due to benign prostatic hyperplasia, neurodegenerative diseases, tumors and other factors, thus becoming a high-frequency population for indwelling urinary catheterization or stent implantation (Xiao et al., 2024; Truzzi et al., 2025). At the same time, advanced age itself is a stage of aggregation of various chronic diseases (e.g., hypertension, diabetes mellitus, chronic kidney disease, cardiovascular and cerebrovascular diseases) (Stucker et al., 2021), which makes this population in a relatively fragile state of immune response when facing the risk of device implantation. In terms of department distribution, patients were highly concentrated in the Department of Urology (31.8%) and ICU (21.4%), accounting for more than half of the total number of patients. This distribution pattern clearly depicts the two main clinical scenarios of device-associated infections: invasive diagnosis and treatment of urinary system diseases and life support for critically ill patients (Ehdaie et al., 2021; Sleziak et al., 2025). The above data suggest that the prevention and control of such infections must become a core link in the quality improvement and infection control of the Department of Urology and ICU. However, after stratification according to the number of underlying diseases, there were no statistically significant differences in gender and age composition among Group A, B and C, indicating that underlying disease burden is a relatively independent variable of age and gender in the study cohort, and its distribution in different demographic subgroups is relatively balanced. This supports the feasibility of using the number of diseases as a universal risk index across populations, and also suggests that age and gender themselves are not the main confounding factors leading to the differences in outcomes among groups in this study cohort.

In-depth analysis of patients’ clinical symptoms reveals a clinically significant pattern. The present study found that although the incidence of fever, urinary irritation symptoms (frequent micturition, urgent micturition, dysuria) and lumbago (lumbosacral soreness, low back pain) showed no statistically significant differences among the three groups, a noteworthy numerical trend was observed. Specifically, the incidence of fever and lumbago presented an increasing trend with the aggravation of underlying disease burden (fever: 35.4% in Group A, 38.1% in Group B, 41.9% in Group C; lumbago: 5.4% in Group A, 11.9% in Group B, 10.8% in Group C). This trend may imply two pathophysiological implications: first, multiple comorbidities may indicate poorer overall health status and immune regulation function (Chang et al., 2024; Zhang et al., 2024), and the body is more likely to initiate a strong systemic inflammatory response when infected, manifested as an increased proportion of fever; second, patients with a heavy burden of underlying diseases may have a weaker local defense mechanism of the urinary system (e.g., increased residual urine caused by neurogenic bladder, urine stasis caused by obstructive diseases (Hou et al., 2025)), and infection is more likely to ascend along the urinary tract, involving the renal pelvis and even renal parenchyma, thus causing more obvious localized lumbago symptoms. Contrary to the trend of fever, the typical urinary irritation symptoms did not increase with the aggravation of disease burden, and even slightly decreased in Group C. This may be because the indwelling catheter continuously drains urine, bypassing the normal physiological process of bladder filling and emptying, thus partially or completely eliminating the typical sensations of frequent micturition and urgent micturition; meanwhile, the long-term mechanical stimulation of the bladder mucosa by the catheter may cause bladder hypoesthesia (Shen et al., 2023; Göransson et al., 2025). Therefore, for patients with indwelling medical devices and a heavy burden of underlying diseases, clinicians must not relax their vigilance against urinary tract infections because the patients lack typical urinary irritation symptoms. On the contrary, for such high-risk populations, even if only non-specific systemic discomfort, altered mental status or unexplained elevation of inflammatory indicators occur, urine examinations should be actively performed to avoid delayed diagnosis and treatment. Diagnosing symptomatic urinary tract infection is extremely challenging in patients with indwelling urinary devices, especially those in the ICU. ICU patients are often under sedation, receiving mechanical ventilation, or presenting with neurological impairment, and are therefore unable to report typical urinary symptoms such as dysuria, frequency, and urgency. They may only manifest non-specific clinical signs, including unexplained fever, hypotension, delirium, or elevated inflammatory markers without an identifiable infectious focus (Ling et al., 2023). These manifestations overlap with other common ICU-acquired illnesses, such as catheter-related bloodstream infection, pneumonia, and intra-abdominal infection (Dettori et al., 2023), making the definitive diagnosis of urinary tract infection particularly difficult.

A more fundamental issue is the extremely high prevalence of asymptomatic bacteriuria among patients with long-term indwelling catheters. Studies have demonstrated that nearly all patients develop bacteriuria within 30 days of prolonged urinary catheterization, and the vast majority of these cases are asymptomatic (Kidd et al., 2015). Current guidelines recommend against routine screening or treatment of asymptomatic bacteriuria, except for specific high-risk populations. The rationale is clear: treating asymptomatic bacteriuria cannot reduce the risk of subsequent symptomatic infection; on the contrary, it increases the risk of antibiotic-associated diarrhea, Clostridioides difficile colitis, and the emergence of multidrug-resistant organisms (Barlam et al., 2016). Accordingly, a strict distinction must be made in both clinical practice and research between symptomatic infection requiring antimicrobial therapy and asymptomatic bacteriuria that does not warrant treatment.

Another complicating factor is the polymicrobial nature of device-associated urinary tract infection (DA-UTI), particularly in patients with indwelling catheters placed for weeks or months. Biofilms formed on the inner and outer surfaces of catheters act as a reservoir for diverse bacteria and fungi, including enterococci, *Escherichia coli*, *Klebsiella pneumoniae*, *Pseudomonas aeruginosa*, and various *Candida* species (Ramstedt et al., 2019). These microorganisms interact through synergistic or antagonistic effects, jointly altering antimicrobial susceptibility profiles and rendering empirical antibiotic selection highly unpredictable.

In the present study, the isolation rate of *Candida* was observed to rise with increasing burden of underlying comorbidities, further highlighting the clinical relevance of polymicrobial biofilms. Patients with multiple comorbidities may be particularly susceptible to fungal overgrowth, and empirical regimens lacking antifungal coverage are likely to fail.

In summary, the aforementioned diagnostic and therapeutic challenges—including identification of symptomatic infection among non-communicative ICU patients, avoidance of unnecessary treatment for asymptomatic bacteriuria, and polymicrobial biofilm formation on long-term indwelling devices—collectively contribute to the high failure rate of empirical therapy and the dissemination of antimicrobial resistance. Traditional risk stratification strategies fail to fully address these concerns, and dedicated attention should be paid to these issues in future research.

In terms of etiological composition, *Escherichia coli* (41.6%) was the absolutely dominant pathogen among the isolated pathogens in this study, followed by *Klebsiella pneumoniae*, *Candida albicans* and *Enterococcus faecium*. This composition is consistent with most reports on hospital-acquired or device-associated UTIs (Kranz et al., 2020; Shen et al., 2023), reflecting that intestinal-derived Gram-negative bacilli, especially *Escherichia coli*, still maintain a strong colonization and survival advantage in the biofilm niche provided by indwelling devices (Wi and Patel, 2018). However, there were no statistically significant differences in the constituent ratios of major pathogens among the groups with different underlying disease burdens. The reason for this result may be that indwelling medical devices themselves, as an extremely strong exogenous risk factor, partially mask the differential selection effect of different underlying disease backgrounds on pathogen colonization. Device implantation creates a physical basis for biofilm formation, providing a relatively homogeneous colonization and invasion environment for common intestinal flora (especially *Enterobacteriaceae*) (Arciola et al., 2018). Nevertheless, a clear trend was that the isolation rate of *Candida* species showed a steady upward trend with the increase in the number of underlying diseases (rising from 13.6% in Group A to 22.1% in Group C). Although this trend did not reach a statistically significant level in the sample size of this study, its clinical significance cannot be ignored. It strongly suggests that the accumulation of comorbid states jointly creates a microenvironment more conducive to fungal colonization and proliferation through a variety of pathophysiological pathways, such as dysbiosis caused by more frequent exposure to broad-spectrum antibiotics (Altveş et al., 2020), hyperglycemic urine environment caused by metabolic diseases such as diabetes mellitus (Ahmad et al., 2020), and immunosuppressive state associated with chronic diseases (Hebert et al., 2021). Therefore, when managing patients with indwelling medical devices and multiple underlying diseases, especially those with poor response to initial antibacterial therapy, clinicians must include candidiasis in the primary differential diagnosis.

The core contribution of this study is to systematically quantify and confirm the significant association between underlying disease burden and antimicrobial resistance of major Gram-negative pathogens in the specific context of indwelling medical devices, and to construct a predictive bridge from host characteristics to microbial drug resistance. In-depth analysis of the drug resistance pattern of *Escherichia coli* reveals the most clinically alarming finding: underlying disease burden is a key independent predictor of its resistance to fluoroquinolones. Compared with Group A patients without underlying diseases, the isolated strains from patients with ≥2 underlying diseases (Group C) had a sharp increase in the risk of resistance to ciprofloxacin, levofloxacin, moxifloxacin and norfloxacin, among which the risk of ciprofloxacin resistance increased by 7.69 times. The markedly increased fluoroquinolone resistance in patients with multiple comorbidities and indwelling devices (Group C) can be explained by a combination of mechanisms. On one hand, patients with multiple underlying diseases often receive frequent antibiotic courses for their chronic conditions (Kang et al., 2006; Sekoni et al., 2022), which generates strong selective pressure and promotes resistance (Zhong et al., 2024). On the other hand, the indwelling device itself acts as a foreign body, providing a surface for bacterial colonization and biofilm formation (Yamada and Kielian, 2019). Biofilms reduce antibiotic penetration and efficacy, thereby further driving the development of resistance. This explains why even patients with a single comorbidity (Group B) already show significantly higher resistance to levofloxacin and norfloxacin, and why the risk escalates further in Group C (OR = 7.69 for ciprofloxacin).

Crucially, for levofloxacin and norfloxacin, even patients with only one underlying disease (Group B) had a significantly higher resistance risk than Group A. Fluoroquinolones have broad-spectrum activity against Gram-positive and Gram-negative aerobic and anaerobic bacteria, with good tissue and intracellular permeability, high bioavailability and good oral tolerance, and have been widely used in the treatment and prevention of UTIs (McGee et al., 2019). However, the extensive use of fluoroquinolones may also lead to the widespread presence of their resistance genes in the medical environment and the patient’s intestinal flora. The data of this study indicate that under the current antimicrobial resistance situation, empirical selection of fluoroquinolones as initial therapy for patients with DA-UTIs complicated with underlying diseases (especially multiple comorbidities) is equivalent to bearing an extremely high risk of clinical failure.

In addition, the resistance risk to ceftizoxime was also significantly increased in Group C, suggesting that with the aggravation of comorbid burden, the problem of resistance to some third-generation cephalosporins also needs to be vigilant. However, it is somewhat reassuring that carbapenems, aminoglycosides, polymyxins and tigecycline have maintained high susceptibility in all groups, guarding a key therapeutic line for coping with possible infections caused by multidrug-resistant Gram-negative bacteria.

In the analysis of *Klebsiella pneumoniae*, we observed another pattern of the association between underlying disease burden and antimicrobial resistance. Compared with patients without underlying diseases, the isolated strains from patients with one underlying disease (Group B) had a significantly increased risk of resistance to cefotetan, doripenem and amikacin. This finding is particularly alarming because cefotetan is effective against some extended-spectrum β-lactamase (ESBL)-producing strains (Stewart et al., 2021), and under specific susceptibility test conditions, doripenem and amikacin can be used as important alternative options for the treatment of infections caused by carbapenem-resistant Enterobacteriaceae (CRE) (Kranz et al., 2024). The early increase in the risk of resistance to these key drugs in patients with only one underlying disease strongly suggests that this patient group may have become a high-risk population for early colonization or infection with drug-resistant bacteria. This heightened risk in Group B could be driven by several intertwined factors. Patients with even a single significant underlying disease are likely to have had repeated healthcare exposures, creating opportunities for colonization with multidrug-resistant *Klebsiella pneumoniae* harboring extended-spectrum β-lactamases (ESBLs) and/or plasmid-mediated AmpC enzymes, which frequently hydrolyze cephamycins like cefotetan (Chaudhary et al., 2023). Concurrently, the parallel increase in resistance to doripenem and amikacin may reflect the clonal expansion of carbapenem-resistant *K. pneumoniae* (CRKP) strains that co-carry aminoglycoside resistance determinants, such as 16S rRNA methylases, within healthcare environments (Takei et al., 2022). The clustered loss of susceptibility across three distinct antibiotic classes—carbapenems, aminoglycosides, and cephamycins—strongly suggests the circulation of high-risk clones (e.g., ST258) that accumulate multiple resistance mechanisms, thereby limiting therapeutic alternatives early in the disease course (Baker et al., 2018). At the same time, the overall high background resistance rate of *Klebsiella pneumoniae* to carbapenems in the data of this study once again confirms the global challenge brought by the prevalence of CRE (Nordmann et al., 2011; Akeda, 2021).

The results of this study have profound clinical implications and clear practical guiding value. First, it constructs a simple and practical clinical risk assessment framework. When facing urinary tract infections in patients with indwelling medical devices, clinicians should quickly assess the number of underlying diseases before obtaining antimicrobial susceptibility test results. For patients with low disease burden (Group A), conventional antibiotics can be carefully selected with reference to local epidemiology; for patients with moderate and high disease burden (Group B and C), especially Group C, it is necessary to consider that the infected strains may have a high risk of resistance to fluoroquinolones and some cephalosporins. Initial empirical therapy should prioritize antibiotics with broader coverage and lower resistance rates (e.g., β-lactamase inhibitor combinations), and the principle of etiological testing before medication should be strictly implemented. Second, this study provides a new entry point for the scientific management of antibiotics in hospitals. Underlying disease burden can be incorporated into the prescription review and early warning system, and more stringent medication monitoring can be implemented for patients with high disease burden to promote precise treatment and delay the emergence of antimicrobial resistance. Finally, patients with indwelling medical devices and multiple underlying diseases should be regarded as a super key population for infection prevention. While strengthening the standardized management of indwelling devices, the overall treatment of their primary chronic diseases should be actively optimized to improve the host’s intrinsic defense capacity.

The preserved susceptibility to carbapenems, aminoglycosides, polymyxins and tigecycline, particularly against *Escherichia coli*, may partly be attributed to antimicrobial stewardship principles, which recommend restricting the use of broad-spectrum antibiotics to reduce selective pressure and preserve their activity (Chaudhary et al., 2023). Moreover, polymyxins and tigecycline, as last-resort antibiotics, are typically reserved for confirmed multidrug-resistant infections, thereby limiting the emergence of resistance (Jeong et al., 2025). Nevertheless, this reassuring profile must be interpreted with caution and should not be generalized across all pathogens: the elevated doripenem resistance observed specifically in *Klebsiella pneumoniae* from Group B serves as a critical early warning that carbapenem susceptibility can erode more rapidly in certain species within comorbid populations. Sustained, species-stratified surveillance of these key agents remains essential.

This study also has several limitations. The single-center retrospective design may limit the generalizability of the conclusions; the sample size (especially the sample size of Group C) may affect the statistical power of some subgroup analyses (e.g., clinical symptoms, certain resistance rates). The small sample size of Group C (n=93) is a particular limitation; several observed trends, such as the increasing isolation rate of *Candida* species with higher comorbidity burden, did not reach statistical significance, which may be attributable to insufficient power rather than absence of a true effect. Using the number of diseases as an indicator of disease burden fails to reflect the heterogeneous impact of different disease types and severity. This study did not collect data on prior antibiotic exposure or frequency of healthcare contact, which may be collinear with underlying disease burden and affect resistance. Thus, the observed increase in resistance risk might partially reflect higher healthcare exposure rather than an independent effect of comorbidity burden. Future prospective studies should collect these variables for multivariable adjustment. Furthermore, although this study strictly defined symptomatic infection according to EAU guidelines and excluded asymptomatic bacteriuria, the retrospective design relied on medical records, which may introduce information bias and cannot completely rule out the possibility that some cases of asymptomatic bacteriuria were inadvertently included. In addition, this study did not systematically analyze the polymicrobial nature of DA-UTIs (e.g., the clinical significance of coexistence of multiple pathogens in long-term indwelling catheters), which could be an important factor contributing to treatment failure in some patients and deserves further investigation in future prospective studies.

### 5 Conclusions

This study reveals that underlying disease burden (measured by the number of comorbid diseases) is an independent and key factor predicting a significant increase in the risk of *Escherichia coli* resistance to fluoroquinolones in patients with device-associated urinary tract infections. With the increase in the number of underlying diseases, the incidence of candidiasis shows an upward trend, and the clinical manifestations tend to be atypical, while carbapenems, aminoglycosides and polymyxins still maintain high susceptibility.

## Data Availability

The original contributions presented in the study are included in the article. Further inquiries can be directed to the corresponding author.
